# Addressing the Social and Medical determinants of Health for Safe Medicines Use

**DOI:** 10.1093/geroni/igab046.2377

**Published:** 2021-12-17

**Authors:** June Simmons, Raymond Woosley, Ester Sefilyan

**Affiliations:** 1 Partners in Care Foundation, San Fernando, California, United States; 2 CredibleMeds, Inc., Oro Valley, Arizona, United States

## Abstract

Both social and medical factors can negatively affect health outcomes, especially in vulnerable populations. To address these two types of factors in a hospital post-discharge population, two non-profit organizations collaborated to combine their novel decision support programs to address the question: could combined programs have greater potential for improved health outcomes? HomeMeds (HM), a social health program in which trained social services staff make home visits to vulnerable clients, was combined with MedSafety Scan (MSS), a medical health, clinical decision support tool. Data captured in the home visits were entered into the HM and MSS programs to detect those patients at greatest risk of adverse health outcomes due to medications. Patients received a post-discharge home visit by trained social services staff. The number of drugs reported as being taken was on average 10.4, which was less than prescribed at discharge in 62% of patients. Both programs detected serious risk of medication-induced harm, mostly from different causes such as drug-drug interactions or for use not recommended in older adult. Combined analysis of data from two novel decision support programs yielded complementary findings that together address both medical and social determinants of health. These have the potential to reduce medication-induced harm, costly re-hospitalization and/or emergency room visits and support the further evaluation of this combined approach in other vulnerable populations such as the seriously mentally ill, frail, those confined to home, opioid-dependent or otherwise impaired.

